# Gold-Nanoparticles Reflectance Discriminates Benign from Malignant Salivary Gland Neoplasms

**DOI:** 10.3390/jcm14051672

**Published:** 2025-03-01

**Authors:** Shiran Sudri, Irit Allon, Ilana Kaplan, Abraham Hirshberg, Dror Fixler, Imad Abu El-Naaj

**Affiliations:** 1 Department of Oral and Maxillofacial Surgery, Tzafon Medical Center, affiliated with Azrieli Faculty of Medicine, Bar Ilan University, Poriya 1520800, Israel; iabu@poria.health.gov.il; 2 Institute of Pathology, Barzilai Medical Center, affiliated with Ben Gurion University of the Negev, Beer Sheba 84105, Israel; allonirit1@gmail.com; 3 Department of Oral Pathology and Oral Medicine and Maxillofacial Imaging, School of Dental Medicine, Tel Aviv University, Tel Aviv 69978, Israel; dr.ilanakaplan@gmail.com (I.K.); hirshmd@tauex.tau.ac.il (A.H.); 4 Sheba Cancer Research Center, The Chaim Sheba Medical Center at Tel-Hashomer, affiliated with Sackler School of Medicine, Tel-Aviv University, Tel-Aviv 52621, Israel; 5 Faculty of Engineering and the Institute of Nanotechnology and Advanced Materials, Bar Ilan University, Ramat Gan 5290002, Israel; dror.fixler@biu.ac.il; 6 The Azrieli Faculty of Medicine in the Galilee, Bar-Ilan University, Safed 1311502, Israel

**Keywords:** salivary gland malignancies, head and neck cancer, gold-nanoparticles, nanophotonics, GNS, EGFR, hyperspectral microscopy, immunohistochemical analysis

## Abstract

**Objectives:** This study aimed to assess the effectiveness of gold nanoparticles conjugated with anti-EGFR monoclonal antibodies (GNPs-EGFR) in distinguishing between benign and malignant salivary gland tumors. **Methods:** A total of 49 oral salivary gland tissue samples were analyzed, including 22 malignant salivary gland tumors (MSGTs), 15 benign salivary gland tumors (BSGTs), and 12 control samples. For each sample, three 5 μm consecutive tissue sections were prepared. The first section was stained with hematoxylin and eosin (H&E) to confirm the diagnosis, the second was immunohistochemically stained for anti-EGFR, and the third was treated with GNPs-EGFR followed by hyperspectral microscopy to analyze the reflectance spectrum. **Results:** Reflectance intensity was significantly higher (*p* < 0.001) in MSGTs compared to BSGTs and controls, with intensity levels increasing alongside tumor grade. The average hyperspectral reflectance values were strongly correlated with the GNPs-EGFR immunohistochemical score and varied significantly between subgroups (*p* < 0.001). **Conclusions:** GNPs-EGFR reflection measurements effectively differentiate MSGTs from BSGTs with high sensitivity. This diffusion–reflection technique holds potential as a valuable tool for tumor detection, surgical margin assessment, and intraoperative identification of residual disease in salivary gland tumors.

## 1. Introduction

Salivary gland tumors are uncommon, representing only 5% of head and neck tumors [1,2]. Diagnosing these tumors is challenging due to their rarity, varied clinical presentations, and diverse histological characteristics. Regarding salivary gland tumors, the oral cavity is the second most frequently affected site after the parotid gland [3,4]. Distinguishing between benign salivary gland tumors (BSGTs) and malignant salivary gland tumors (MSGT) is particularly difficult because of overlapping clinical features, underscoring the need for advanced diagnostic tools [4]. GNP-EGFR use for bio-imaging suspected malignant salivary gland tumors may enhance tumor identification.

Nanomedicine has gained significant attention in recent years, particularly the use of nanoparticles for diagnostic purposes and targeted drug delivery to cancerous tissues [5,6,7,8]. Nanophotonics, which exploits the light absorption properties of gold nanoparticles (GNPs) in the red to infrared spectrum, offers a promising avenue for cancer detection and imaging [9]. GNPs conjugated with anti-epidermal growth factor receptor (GNPs-EGFR) monoclonal antibodies selectively bind to cancer cells with elevated EGFR expression [10,11,12]. Recent studies utilizing GNPs-EGFR have demonstrated significant differentiation between malignant and benign oral lesions [9,10,11,12].

Increased EGFR expression has been observed in malignant compared to benign salivary gland tumors, correlating with tumor grade [13,14,15,16,17]. The superficial location of these tumors and their high EGFR expression levels make them ideal candidates for nanophotonic methods, enabling the detection of GNPs-EGFR reflections on tumor cells.

The objectives of the present study were to evaluate the detection sensitivity of GNPs-EGFR in a subset of human salivary gland tumors for discriminating benign from malignant tumors with histopathology as a gold standard. The reflectance spectra were captured using hyperspectral imaging microscopy.

## 2. Materials and Methods

Archival cases, previously diagnosed as malignant and benign salivary gland tumors, as well as normal salivary glands, were selected from the archives of the oral and maxillofacial surgery departments at Tzafon Medical Center, Sourasky Medical Center, and Barzilay Medical Center.

The study was approved by the Institutional Review Board of Poriya Medical Center (approval # POR-0011-21), Bazilay Medical Center (approval # BRZ-0111), and the Tel-Aviv Sourasky Medical Center (approval # 0158-18-tlv). The study was performed in accordance with the Declaration of Helsinki, seventh revision (2013). All cases were anonymous, and informed consent was not required. Data concerning age, gender, and salivary gland involvement were recorded.

The cases were divided according to histopathologic diagnosis based on the 5th edition of the World Health Organization (WHO) classification of head and neck tumors [18]. The samples originated from both minor and major salivary glands. The original slides were reviewed, and the diagnosis was confirmed by two of the researchers, both certified oral pathologists (A.H., I.A.). Only cases with definitive diagnoses based on histopathology were included.

From each paraffin-embedded block, three consecutive 5 μm sections were cut on a glass slide. The first was stained with hematoxylin and eosin (H&E) to confirm the diagnosis, the second was used for an immunohistochemical stain for anti-EGFR (NBP1-84814-25ul, Novus Biologicals, Centennial, CO, USA), and an unstained slide was submitted for hyperspectral imaging. The area of interest (AOI) was marked on the H&E-stained slide and copied exactly on the other slides; the hyperspectral imaging and the immunohistochemical measurements were performed only within the AOI.

### 2.1. GNPs Fabrication and Targeting

Self-fabricated gold nanospheres (GNSs) conjugated with anti-EGFR monoclonal antibody Cetuximab (GNS-EGFR) at a concentration of 6 mg/mL were used. Spectrophotometer analysis determined an extinction peak of 530 nm for the GNS-EGFR. GNSs were prepared following the Enüstün and Turkevich method [19], and their uniformity and size were confirmed using a transmission electron microscope. Polyethylene glycol (PEG) was applied to prevent aggregation, and bioconjugation to anti-EGFR antibodies was achieved using the Lvov polystyrene sulfonate method [20]. Bioconjugation was validated using zeta potential and dynamic light scattering measurements.

### 2.2. Hyper Spectral Imaging System

**Figure 1:**
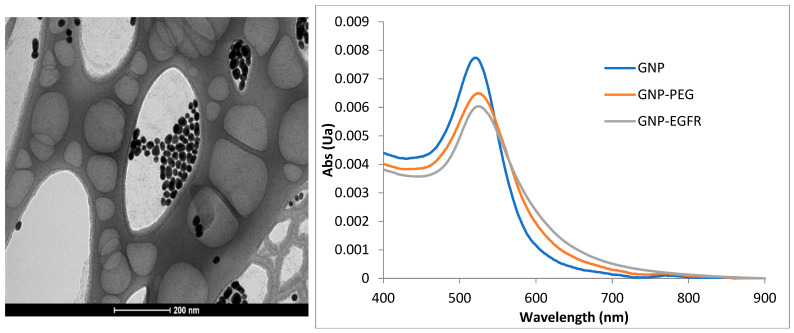
Transmission electron microscopy (TEM) image of the self-fabricated GNS, sized~20 nm, and the peak of the GNS before and after conjugation to PEG and EGFR.

Reflectance measurements were conducted using a hyperspectral imaging system (Nuance, CRi, MA) equipped with a halogen illuminator, a 32-bit ultrasensitive CCD camera detector, and a 40× objective lens. Images were acquired at 530 nm using Nuance software, version 2.1. Reflectance intensities were analyzed after subtracting background and glass spectra. Slides were scanned before and after applying GNS-EGFR for control comparisons (Figure 1).

### 2.3. Immunohistochemistry

Immunohistochemical stain for anti-EGFR (NBP1-84814-25ul, Novusbio) was used according to the protocol of the manufacturer. Tumor cells were identified in each spot by comparison with the H&E-stained consecutively cut.

EGFR expression was evaluated semi-quantitatively based on staining intensity (0, 1+, 2+, 3+) and the fraction of positive tumor cells on the histological slides. Only cells with membranous staining were scored. A composite score was calculated as follows [15]:

0: No staining or membranous staining in <10% of tumor cells.
1: Faint, incomplete membranous staining in ≥10% of tumor cells.
2+: Weak to moderate membranous staining in ≥10% of tumor cells.
3+: Strong membranous staining in ≥10% of tumor cells.

### 2.4. Statistics

The main goal of the analysis was to investigate the power of GNS-EGFR reflectance measurements in discriminating between normal salivary glands and benign and malignant salivary gland tumors.

Hyperspectral measurements were taken from the AOI of each slide and sample and are therefore not homogenous and nonlinear mixed effect models, which is a standard method for repeated measured data that cannot be used. Instead, we defined two intensity indices for each case, maximum and average. We performed analyses for both values; however, the maximum value is more important.

The status of the patient was defined as A for controls (normal salivary gland), B for BSGT, and C for MSGT.

Due to a relatively small sample size and skewed distribution of intensity, we paid attention to the dependence of the results on the method of analysis.

Cuzick’s non-parametric test for trend to test the different diagnoses by three groups and also by nine subgroups.

Mann–Whitney and *t*-test to compare between the three groups (normal vs. BSGT and BSGT Vs MSGT).

Mann–Whitney and *t*-test tests to compare between the EGFR immunohistochemical grades in accordance with hyperspectral microscopy results (healthy vs. BSGT and BSGT vs. MSGT).

We investigated the distribution of the hyperspectral imaging and defined the best cut-point. The sensitivity and specificity are presented for this cut-point. Discrimination ability between classes of maximum or average intensity was tested using exact logistic regression and receiver operating characteristic (ROC) analysis.

The analysis was performed using STATA 16 SE software. All tests were two-sided. *p*-values under 0.05 were described as significant.

## 3. Results

A total of 49 cases were examined. Table 1 summarizes the various groups: Group A included 12 cases of normal salivary glands; Group B, BSGT, included 15 cases of pleomorphic adenoma; Group C, MSGT, included 22 cases divided into six subgroups: squamous cell carcinoma, polymorphous low-grade carcinoma, salivary duct carcinoma, mucoepidermoid carcinoma, adenoid cystic carcinoma, and acinic cell carcinoma.

A total of 19 of the cases originated from minor salivary glands, and 30 originated from major salivary glands.

The most effective discriminating statistic was derived from the maximum intensities measured by hyperspectral microscopy, reflecting the maximum absorption values of GNP-EGFR. The optimal cut-off point for this statistical analysis was determined to be 100 arbitrary units (Au).

Immunohistochemical expression of EGFR score was evaluated using intensity (0, 1+, 2+, 3+) and the fraction of positive cells at the AOI. The 0 and 1 intensities were combined and referred to as 1 due to the low number of samples ranked as 0 and the clinical meaning of the results (both refer to the control group).

**Figure 2:**
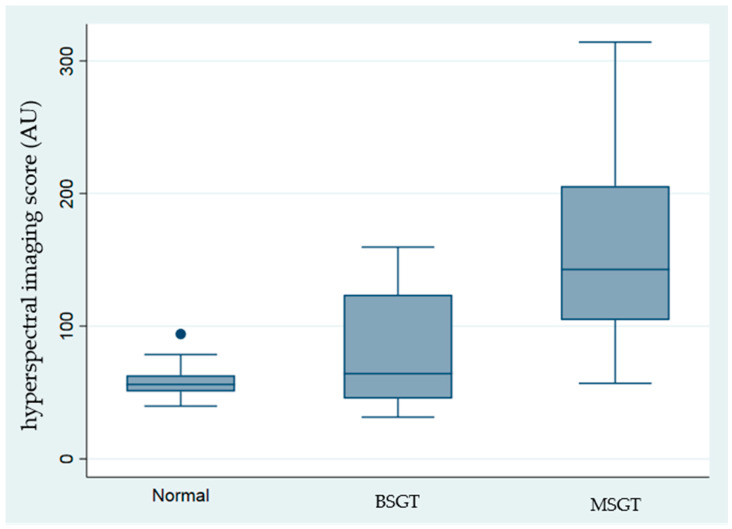
Hyperspectral imaging highest intensities (Au) for each group.

Table 1 and Figure 2 present the recorded hyperspectral values alongside the EGFR immunobiological score. The highest values were recorded in MSGT, mainly in the adenoid cystic carcinoma and acinic cell carcinoma subgroups (203.3 ± 50.71 and 212.29 ± 62.29, respectively).

The average EGFR immunohistochemical score was 3 ± 0 for the adenoid cystic carcinoma subgroup and 2.67 ± 0.47 for acinic cell carcinoma. The lowest values were measured in the normal salivary gland group with hyperspectral values of 50.68 ± 16.29 and an EGFR score of 0.83 ± 0.55, reflecting the low concentration of EGFR. Intermediate values were recorded in pleomorphic adenoma 69.79 ± 27.97, 1.2 ± 0.4, respectively. The mucoepidermoid carcinoma group is composed of high-grade and low-grade tumors. Due to the low number of samples, the two groups were combined. The mean hyperspectral values were 114.57 ± 48.77, and the EGFR score was 2.14 ± 0.83.

Figure 2, the Cuzick non-parametric test of trend, revealed that significantly high intensities were recorded in the malignant salivary gland tumors, compared with benign and control groups (*p* < 0.001).

Mann–Whitney and *t*-tests were performed to compare between all groups (Table 2 and Table 3). Both tests found significant differences comparing normal salivary glands (group A) and MSGT (group C) (*p* < 0.005). Comparing MSGT with normal salivary glands revealed a sensitivity of 79% and a specificity of 100%. Comparing between MSGT (group C) and the other groups (normal salivary gland—A, and BSGT—B), the sensitivity was 79% and specificity 83% (*p* < 0.01).

The comparison between normal salivary gland tumor (A) and BSGT (B) was insignificant (*p* > 0.05). Similar results were found comparing the differences in highest intensity scores and the immunohistochemical EGFR staining grade.

The differences between groups A vs. B and B vs. C were significant even after Bonferroni adjustment for multiple comparisons (*p*-values of *t*-test and Mann–Whitney test were less than 0.001) (Table 2 and Table 3).

The average hyperspectral values strongly correlate with the EGFR immunohistochemical score and differ between the subgroups. All paired comparisons had *p* < 0.001 even after corrections for multiple comparisons.

**Figure 3:**
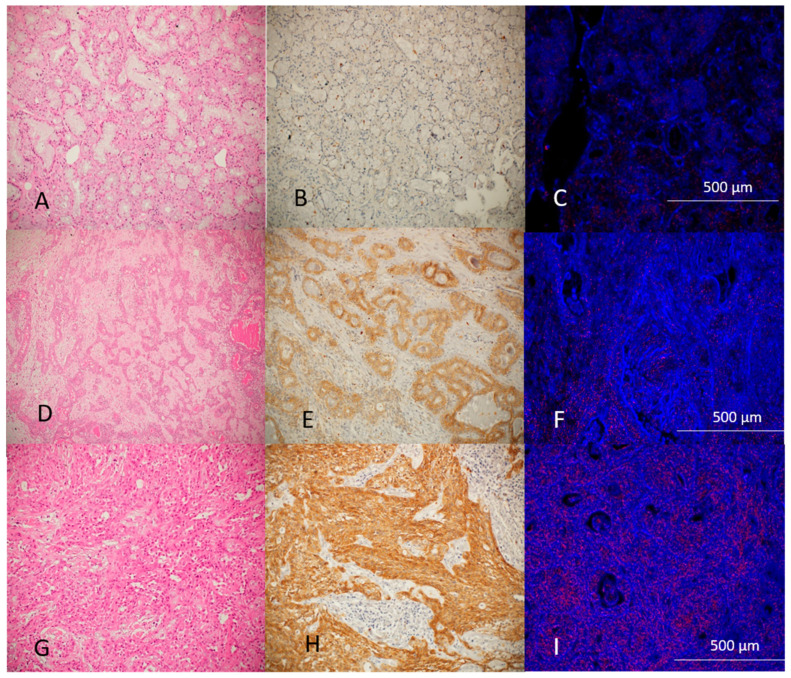
Histological, immunohistochemical, and hyperspectral imaging of a normal salivary gland, benign tumor, and malignant tumor. (**A**–**C**) Normal salivary gland; (**A**) Original magnification ×100 hematoxylin and eosin; (**B**) Original magnification ×100 EGFR immunohistochemical stain; (**C**) Hyperspectral microscopy imaging original magnification ×200; (**D**–**F**) Benign tumor—pleomorphic adenoma; (**D**) Original magnification ×100 hematoxylin and eosin; (**E**) Original magnification ×100 EGFR immunohistochemical stain; (**F**) Hyperspectral microscropy imaging original magnification ×200; (**G**–**I**) Malignant tumor—mucoepidermoid carcinoma; (**G**) Original magnification ×100 hematoxylin and eosin; (**H**) Original magnification ×100 EGFR immunohistochemical stain; (**I**) Hyperspectral microscopy imaging original magnification ×200.

Figure 3 demonstrates the histological, immunohistochemical, and hyperspectral imaging of normal salivary glands, benign tumors, and malignant tumors. GNP-EGFR are marked as red dots concentrated in higher amounts at MSGT as well as high intensity of immunohistochemical stain.

**Figure 4:**
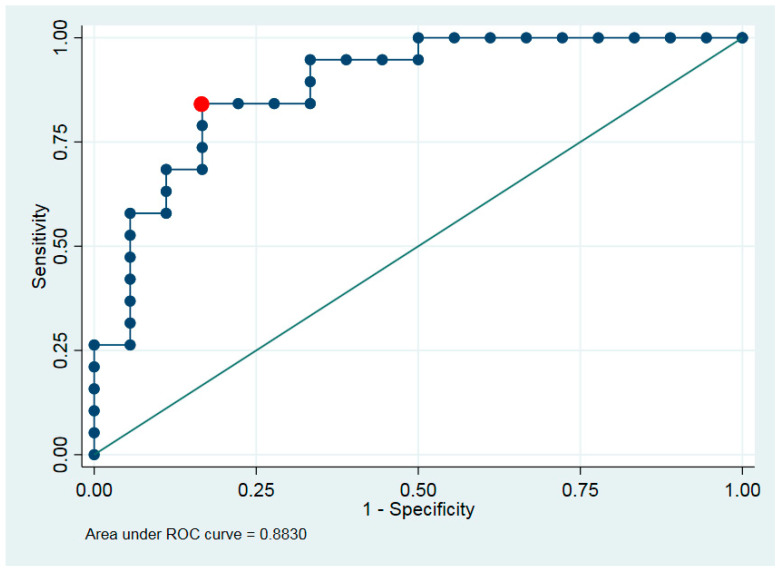
ROC analysis for separation group A—normal salivary gland; group B—BSGT; and group C—MSGT. Exact sensitivity is marked in red.

Discrimination ability was characterized by area under ROC curve (AUC) (Figure 4).

## 4. Discussion

The ability to distinguish between benign and malignant salivary gland tumors preoperatively is essential for determining the most appropriate surgical approach, particularly for small intraoral tumors. Although imaging modalities such as ultrasonography, CT, and MRI have been explored, their diagnostic value is often limited due to challenges in accurately defining tumor margins and tissue characteristics [21,22,23]. For intraoral salivary gland tumors, clinical examination remains the primary diagnostic approach; however, histological confirmation is mandatory as clinical appearance alone cannot reliably predict tumor behavior.

Fine-needle aspiration (FNA) is a commonly employed method for diagnosing salivary gland masses, especially for major glands like the parotid and submandibular glands. While FNA offers the advantages of minimal invasiveness and low complication risk, its diagnostic accuracy can be inconsistent. Sensitivity and specificity rates vary widely across studies, with recent meta-analyses reporting sensitivity as low as 65% and specificity around 97% [24,25,26]. FNA also lacks the ability to assess critical histological factors such as tumor grade, lymphatic involvement, and perineural invasion, which are crucial in determining the extent of surgical resection and the need for adjuvant therapy [27].

The present study demonstrates the power of direct GNP-EGFR reflectance measurements as a novel method for discriminating benign from malignant salivary gland neoplasms and even suggesting a specific tumor detection method. Significantly high intensity, corresponding with the maximum absorption values of the GNS-EGFR, has been found in malignant neoplasms compared with benign tumors. To strengthen our results, due to the rarity and diversity of the lesion, we used different statistical analysis methods.

The use of optical techniques for tumor diagnosis is a relatively new frontier in medicine. These methods leverage the unique light absorption properties of GNPs in the red-to-infrared spectrum, which enables targeted imaging of cancer cells. GNP-EGFR specifically binds to cells with high EGFR expression, making it a powerful tool for distinguishing malignant tumors [9,10,11,12]. Our previous studies demonstrated the efficacy of GNP-EGFR in differentiating oral squamous cell carcinoma from less severe lesions, and the current findings extend this potential to salivary gland tumors [9,11,12].

An increased number of reports have been published in recent years on the expression of epidermal growth factor receptor in salivary gland tumors due to the potential for treatment with targeted therapy, particularly in aggressive salivary duct carcinomas [13,14,15,16,17]. Using immunohistochemistry and FISH analysis performed on surgical material disclosed an increased expression of EGFR in malignant as opposed to benign salivary gland tumors, correlating with the grade of the tumor [13,14,15,16,17]. The results of the present study using semi-quantitative analysis of the immunohistochemical stain with anti-EGFR agree with previous reports [10,11,12], revealing a significant statistical difference between malignant and benign salivary gland tumors with a significant correlation with the nanophotonic results. The diversity inside the subgroups can be referred to as the histological grade of the tumor. The hyperspectral values for high-grade malignancy were higher than in low-grade tumors.

Reflectance measurements of GNP-EGFR attached to tumor cells have great potential in several clinical aspects. It may serve as an objective method augmenting the histopathologic diagnosis, as has been shown in the present study. The use of GNP-EGFR for bio-imaging of suspected malignant salivary gland tumors may enhance tumor identification. Added GNPs as contrast agents for X-ray, computed tomography (CT), surface-enhanced Raman scattering, and photoacoustic tomography (PAT) have been proven to be useful in imaging body structures, providing a relatively high spatial resolution [7,28,29]. In accordance with our latest research in the field of oral cancer [12], using the in vivo diffusion–reflection method may provide a highly sensitive tool for non-invasive clinical tumor detection in discriminating benign from malignant salivary gland tumors, determining the surgical margins accurately, and detecting residual disease in the surgical bed intraoperatively, and this study can be used as a proof of concept for that.

The limitations of our method derive from its reliance on nanoparticles coated with antibodies, as not all salivary gland malignancies express the same antibodies or share identical molecular markers [30,31]. Therefore, a panel of antibodies is required for the specific and definitive diagnosis of the tumors. Further advancements and research are essential to enhance the diagnostic capabilities of this approach.

Accurate and early detection of MSGT is a critical challenge. The relatively superficial presence of these lesions and the overexpression of EGFR make them ideal for the use of nanophotonic-based detection.

The development of various nanophotonic technologies for in vivo applications will have a substantial impact on the field of early cancer detection.

## 5. Conclusions

The GNPs-EGFR reflection measurements effectively differentiate malignant from benign salivary gland tumors. We anticipate that employing the diffusion–reflection method in vivo could offer a highly sensitive tool for clinical tumor detection, aiding in the distinction between benign and malignant salivary gland tumors, accurately determining surgical margins, and detecting residual disease in the surgical bed during surgery.

## Figures and Tables

**Table 1:** Hyperspectral imaging highest score (Au) range according to the histological examination.

Group	Histological Examination (Number of Cases)	Hyperspectral Imaging Mean Score (Au) ± SD	Immunohistochemical Score ± SD
Group A—control	Normal salivary gland (12)	50.68 ± 16.29	0.83 ± 0.55
Group B—benign salivary gland tumor	Pleomorphic adenoma (15)	69.79 ± 27.97	1.2 ± 0.4
Group C—malignant salivary gland tumor	Squamous cell carcinoma (4)	134.11 ± 22.3	2.5 ± 0.5
	Polymorphous adenocarcinoma (1)	216.67	3
	Salivary duct carcinoma (3)	132.91 ± 22.2	2.33 ± 0.47
	Mucoepidermoid carcinoma (7)	114.57 ± 48.77	2.14 ± 0.83
	Adenoid cystic carcinoma (4)	203.3 ± 50.71	3 ± 0
	Acinic cell carcinoma (3)	212.29 ± 62.29	2.67 ± 0.47
	Summary	158.65 ± 65.84	2.204 ± 0.82

SD—standard deviation.

**Table 2:** Comparison between group A (control), B (BSGT), and group C (MSGT) (*t*-test and M-W rank test.

Group	Cases	Mean	SD	Median	Q25%	Q75%	*p* (Compared with)		
							A	B	C
Normal (A)	12	50.68	17.02	55.35	49.62	61.69	-	0.21 *	<0.005 *
BSGT (B)	15	69.79	28.95	65.27	45.34	78.49	0.49 ^	-	<0.01 *
MSGT (C)	22	158.65	65.84	148.82	117.47	205.59	<0.001 ^	<0.01 ^	-

* *t*-test, ^ M-W test

**Table 3:** Comparison summary (*t*-test and M-W rank test).

Test	Normal vs. BSGT	Normal vs. MSGT	BSGT vs. MSGT
*t*-test	0.2067	<0.005	<0.01
M-W rank sum *	0.4894	<0.0001	<0.01

* Exact probability.

## Data Availability

The raw data supporting the conclusions of this article will be made available by the authors on request.
